# Replication Kinetics of B.1.351 and B.1.1.7 SARS-CoV-2 Variants of Concern Including Assessment of a B.1.1.7 Mutant Carrying a Defective ORF7a Gene

**DOI:** 10.3390/v13061087

**Published:** 2021-06-07

**Authors:** Alyssa T. Pyke, Neelima Nair, Andrew F. van den Hurk, Peter Burtonclay, Son Nguyen, Jean Barcelon, Carol Kistler, Sanmarié Schlebusch, Jamie McMahon, Frederick Moore

**Affiliations:** 1 Public Health Virology Laboratory, Forensic and Scientific Services, Department of Health, Queensland Government, Coopers Plains, QLD 4108, Australia; Neelima.Nair@health.qld.gov.au (N.N.); Andrew.VanDenHurk@health.qld.gov.au (A.F.v.d.H.); Peter.Burtonclay@health.qld.gov.au (P.B.); Jean.Barcelon@health.qld.gov.au (J.B.); Carol.Kistler@health.qld.gov.au (C.K.); Jamie.McMahon@health.qld.gov.au (J.M.); Frederick.Moore@health.qld.gov.au (F.M.); 2 Public Health Microbiology Laboratory, Forensic and Scientific Services, Department of Health, Queensland Government, Coopers Plains, QLD 4108, Australia; Son.Nguyen@health.qld.gov.au; 3 Public and Environmental Health, Forensic and Scientific Services, Department of Health, Queensland Government, Coopers Plains, QLD 4108, Australia; Sanmarie.Schlebusch@health.qld.gov.au; 4 Pathology Queensland, Royal Brisbane and Women’s Hospital, Herston, QLD 4006, Australia; 5 School of Medicine, Faculty of Medicine, University of Queensland, Herston, QLD 4072, Australia

**Keywords:** SARS-CoV-2, B.1.1.7, B.1.351, SARS-CoV-2 South African variant, SARS-CoV-2 UK variant, ORF7a, culture, replication kinetics

## Abstract

Severe acute respiratory syndrome coronavirus 2 (SARS-CoV-2), the etiological agent of COVID-19, is a readily transmissible and potentially deadly pathogen which is currently re-defining human susceptibility to pandemic viruses in the modern world. The recent emergence of several genetically distinct descendants known as variants of concern (VOCs) is further challenging public health disease management, due to increased rates of virus transmission and potential constraints on vaccine effectiveness. We report the isolation of SARS-CoV-2 VOCs imported into Australia belonging to the B.1.351 lineage, first described in the Republic of South Africa (RSA), and the B.1.1.7 lineage originally reported in the United Kingdom, and directly compare the replication kinetics of these two VOCs in Vero E6 cells. In this analysis, we also investigated a B.1.1.7 VOC (QLD1516/2021) carrying a 7-nucleotide deletion in the open reading frame 7a (ORF7a) gene, likely truncating and rendering the ORF7a protein of this virus defective. We demonstrate that the replication of the B.1.351 VOC (QLD1520/2020) in Vero E6 cells can be detected earlier than the B.1.1.7 VOCs (QLD1516/2021 and QLD1517/2021), before peaking at 48 h post infection (p.i.), with significantly higher levels of virus progeny. Whilst replication of the ORF7a defective isolate QLD1516/2021 was delayed longer than the other viruses, slightly more viral progeny was produced by the mutant compared to the unmutated isolate QLD1517/2021 at 72 h p.i. Collectively, these findings contribute to our understanding of SARS-CoV-2 replication and evolutionary dynamics, which have important implications in the development of future vaccination, antiviral therapies, and epidemiological control strategies for COVID-19.

## 1. Introduction

Throughout history, the rapid emergence of infectious, highly transmissible human respiratory viruses has relentlessly crippled population health and often led to substantial economic instability and loss. Within a short interval of two months, the novel severe acute respiratory syndrome coronavirus 2 (SARS-CoV-2), the viral source of a pneumonia cluster first reported in Wuhan, Hubei Province in late 2019, had invoked escalating case numbers of COVID-19 worldwide and was reported to have caused approximately 2800 deaths in 46 countries [1]. As a result of these dire public health consequences, a pandemic was declared by the World Health Organization (WHO) in March 2020. Now a global threat, the virus has been associated with over 157 million cases and more than 3 million deaths [2].

Analogous with its relative SARS-CoV, SARS-CoV-2 is a member of the genus *β-coronavirus*. While SARS-CoV did not display efficient human-to-human spread, SARS-CoV-2 has higher transmission rates, which have led to the current pandemic [3]. The COVID-19 global health crisis has caused an urgent need for improved understanding of virus–host interactions and the relevance of reported genomic changes, with an emphasis on those suspected of conferring increased viral fitness and/or higher incidence of disease. Several new SARS-CoV-2 genetic variants of concern (VOCs) have been identified [4], which have reported transmissibility rates that surpass previous SARS-CoV-2 variants, including viruses carrying the D614G spike (S) protein amino acid substitution [5]. In particular, VOCs described as the United Kingdom (UK) variant belonging to the B.1.1.7 lineage, the Republic of South Africa (RSA) variant (B.1.351 lineage) and the Brazil variant (B.1.1.28.1 (P.1) lineage) have emerged. These VOCs have multiple concerning amino acid substitutions in the S protein, such as N501Y and E484K, which may be linked to increased virulence and/or immune evasion [4]. Indeed, Davies et. al. have reported new evidence strongly associating increased COVID-19 mortality (approximately 61% higher) with the B.1.1.7 lineage and possible links to increased viral loads in infected patients [6].

The SARS-CoV-2 positive-sense RNA genome of approximately 30,000 nucleotides is well characterized, and sequencing efforts throughout the course of the pandemic have provided unprecedented information on how a virus can evolve as it disseminates around the world [7,8]. Briefly, the 5′ two-thirds of the genome encode the long open reading frames (ORFs) 1a and 1b, which are associated with synthesis of the viral replication proteins and RNA-dependent RNA polymerase (RdRp). Structural and accessory proteins are translated from subgenomic RNAs encoding ORFs incorporated in the 3′ terminal third of the genome [8]. Reasonably conserved among coronaviruses, the key structural proteins, namely S, envelope (E), membrane (M), and nucleocapsid (N), have been targets for the development of many viral diagnostic molecular and serological detection platforms. Utilization of the S protein has largely dominated advancement and production of vaccine and therapeutic agent candidates. Nine less well-characterized SARS-CoV-2 accessory proteins have been predicted from ORFs 3a, 3b, 6, 7a, 7b, 8, 9b, 9c, 10 [8], and more recently 3c [8]. Whilst not widely considered essential for viral structure or replication, these proteins may have influential implications for viral pathogenicity or interaction with host immune responses [9].

Due to its innate positioning on the viral surface and critical role in infection and host immune response mechanisms, the SARS-CoV-2 S protein has been largely prioritized as a genomic surveillance and research target, and its genetic variations and evolution are currently closely monitored. However, genomic-scale, next generation sequencing (NGS) has afforded identification of additional mutations and indels emerging within the genome other than those reported for the S protein which also warrant careful consideration.

Both the B.1.351 and B.1.1.7 VOCs are among the most transmissible SARS-CoV-2 variants reported to date, but little is known about their respective replication dynamics. In the current study, we directly compare the replication kinetics of these VOCs using isolates from adult patients who had traveled to Brisbane, Queensland, Australia from the RSA or the UK, or had been in contact with travelers from the UK between late 2020 and early 2021. Further, we evaluated the replication kinetics of a B.1.1.7 VOC which was shown to have an identical nucleotide sequence to the B.1.1.7 VOC described above, except for a 7-nucleotide deletion in the ORF7a gene. This deletion was verified by NGS and was present in the original patient sample sequence.

## 2. Materials and Methods

### 2.1. Patient Samples, Cell Culture and Virus Isolation

Nasopharyngeal swab samples were obtained from adult patients for routine reference laboratory diagnostics. All samples were collected within 5 days of the recorded onset date of infection. The B.1.351 isolate, QLD1520/2020, was recovered from a person aged over 50 years who had acquired their infection whilst in the RSA. The B.1.1.7 isolates, QLD1516/2021 and QLD1517/2021, were obtained from people aged under 50 years who were infected in the UK and Australia, respectively.

Patient nasopharyngeal swab samples were inoculated onto Vero E6 cell (ATCC, C1008, CRL-1586, Manassas, VA, USA) monolayers as previously described [10]. Passage 2 SARS-CoV-2 stocks were used for the subsequent replication curve kinetic experiments.

### 2.2. Next Generation Sequencing of Virus Isolates and Phylogenetic Analysis

All SARS-CoV-2 isolates were further characterized by massive parallel sequencing using the Nextera XT kit (Illumina, San Diego, CA, USA) for cDNA library construction and paired-end (2 × 150 nucleotides) sequencing using the V2 mid-output kit on a NextSeq 500 machine (Illumina, San Diego, CA, USA) as previously described [11]. Raw sequence reads were processed using Geneious R10 version 10.2.6 software [12], and near-complete genome sequence assembly was achieved by mapping to a reference SARS-CoV-2 2019 genome sequence (strain Wuhan-Hu-1, GenBank accession number MN908947).

To compare the phylogenetic relationship of the SARS-CoV-2 isolates with other sequences obtained in Queensland, Australia between January 2020 and mid-May 2021, a maximum-likelihood (ML) tree was inferred from a complete genome data set of 571 nucleotide sequences (available on GISAID). The multiple sequence alignment was performed using the Multiple Alignment Using Fast Fourier Transform (MAFFT) program, version 7.407 [13], which was masked by in-house scripts (final sequence lengths were 29,903 nucleotides). The regions subjected to masking were based on reported problematic sites [14]. The ML tree was generated using IQTREE2 [15] and the GTR + F + G4 nucleotide substitution model, with the SARS-CoV-2 Wuhan 2019 reference sequence WIV04 (GISAID accession number EPI_ISL_402124) as an outgroup. Tree visualization and plots were produced using ggplot2 and ggtree version 3.1.0 [16] on R version 4.1 [17]. Pangolin version 2.4.2 [18] and pangoLEARN (updated 19 May 2021; https://github.com/cov-lineages/pangolin) were used to assign corresponding lineage designations.

### 2.3. Kinetic Replication Curve Experiments

To evaluate the replication kinetics of the SARS-CoV-2 variants, we infected Vero E6 cell monolayers grown in 6-well microtiter plates at a multiplicity of infection (m.o.i) of 0.01. Cell monolayers were exposed to 500 µL of virus inoculum per well, incubated at 37 °C for 1 h, before the inoculum was removed and cells were washed 3 times with sterile phosphate buffered saline (PBS). After the final wash, cell monolayers were re-fed with fresh Opti-MEM^™^ reduced serum medium supplemented with 0.2% bovine serum albumin (Catalogue number 15260037, ThermoFisher Scientific, Seventeen Mile Rocks, QLD, Australia) and antimicrobials (Catalogue number 15240062, ThermoFisher, Seventeen Mile Rocks, QLD, Australia), and incubated at 37 °C. The replication of each isolate was assessed in triplicate at the time points 0, 4, 8, 12, 16, 24, 48, and 72 h post infection (p.i.). At each time point, culture supernatants were harvested and clarified to remove cell debris by centrifugation at 2,355 × *g* for 5 min at 4 °C before aliquots were frozen at −80 °C. Viral titers were then determined by a 50% tissue culture infectious dose per milliliter (TCID_50_/mL) assay.

The TCID_50_/mL assay consisted of inoculating 10-fold serial dilutions of each of the virus replicates recovered from the kinetic replication experiments into the wells of 96-well microtiter culture plates which were seeded with confluent monolayers of Vero E6 cells. Plates were incubated for 7 days at 37 °C before assessment of viral infection by observance of cytopathic effect (CPE) and calculation of the TCID_50_/mL titer using the Reed–Meunch algorithm [19].

### 2.4. Statistical Analysis

For each of the timepoints p.i., titers of all the replicates of the 3 viruses expressed as TCID_50_/mL were compared using analysis of variance (ANOVA) and Tukey post-test in GraphPad Version 9.1.0 (GraphPad Software, San Diego, CA, USA).

## 3. Results

### 3.1. Virus Isolation

**Figure 1:**
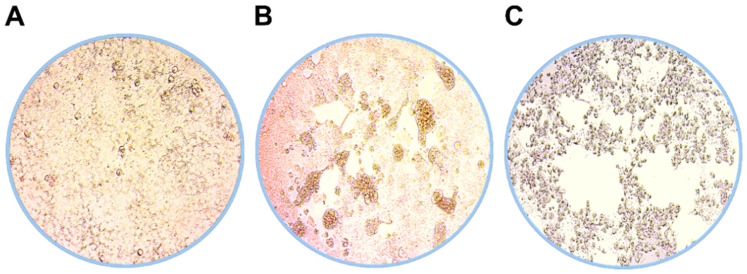
Examples of cell morphology 5 days p.i. in (**A**) uninfected, (**B**) SARS-CoV-2 B.1.1.7, and (**C**) SARS-CoV-2 B.1.351 infected Vero E6 cells representing healthy, low-level CPE with enlarged cells and advanced CPE cultures, respectively.

Virus isolates of the SARS-CoV-2 B.1.1.7 (QLD1516/2021 and QLD1517/2021) and B.1.351 (QLD1520/2020) variants were recovered between 2 and 5 days after inoculation of Vero E6 cells. Examples of CPE and cell morphology observed in uninfected and SARS-CoV-2 infected cells are shown in Figure 1.

### 3.2. Next Generation Sequencing (NGS)

**Figure 2:**
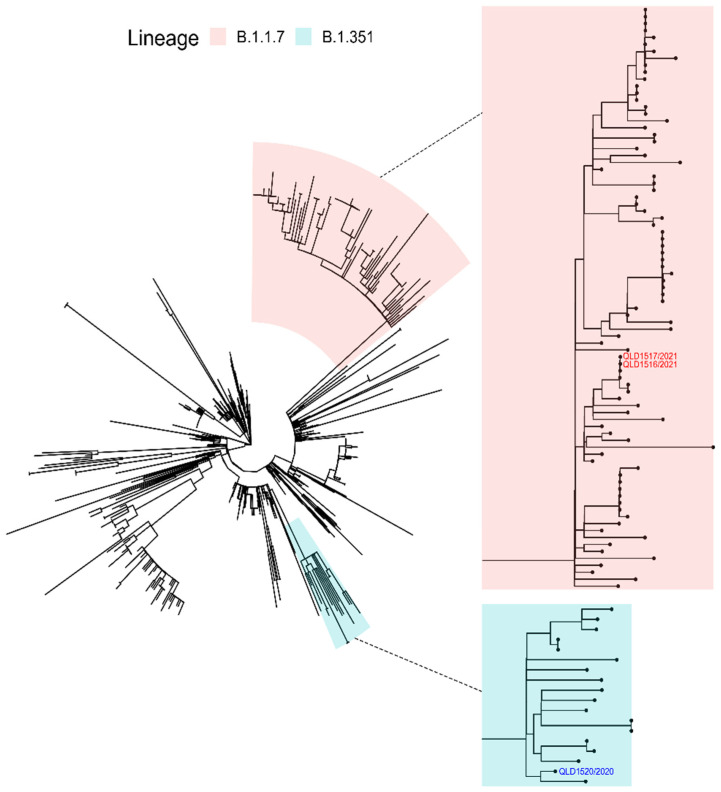
Maximum-likelihood tree inferred from 571 SARS-CoV-2 complete genome nucleotide sequences obtained in Queensland, Australia between January 2020 and mid-May 2021. The B.1.1.7 and B.1.351 lineages, together with the respective SARS-CoV-2 QLD1516/2021, QLD1517/2021, and QLD1520/2020 isolates, are highlighted. The insets attached to dotted lines show the B.1.1.7 and B.1.351 lineage clades at higher resolution.

Resultant near-whole-genome sequences of each of the SARS-CoV-2 VOCs were confirmed to be identical to cognate genomic sequences derived from the original patient samples. The isolate sequences were deposited on GISAID and assigned the following accession numbers: EPI_ISL_1803794 (QLD1516/2021), EPI_ISL_944644 (QLD1517/2021), and EPI_ISL_968081 (QLD1520/2020). An ML phylogenetic tree inferred from 571 Queensland SARS-CoV-2 sequences for the period from January 2020 to mid-May 2021 is given in Figure 2.

**Figure 3:**
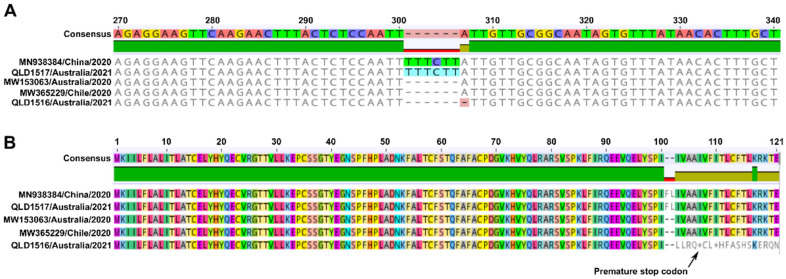
Partial nucleotide (**A**) and complete amino acid sequences (**B**) derived from the SARS-CoV-2 ORF7a gene B.1.1.7 VOCs, QLD1516/2021, and QLD1517/2021. The mutated QLD1516/2021 sequence containing a 7-nucleotide indel is compared with non-mutated sequences of QLD1517/2021 and Wuhan-Hu-1 (GenBank accession number MN938384) together with representative 2020 sequences from Victoria, Australia and Chile containing a 6-nucleotide indel (GenBank accession numbers MW153063 and MW365229, respectively). The position of the putative premature stop codon predicted in the QLD1516/2021 amino acid sequence is indicated by the arrow pointing to an asterisk.

Interestingly, a 7-nucleotide deletion in the ORF7a gene (nucleotides 301-307), which was first identified in the original patient sequence (GISAID accession number EPI_ISL_1300524), was also confirmed in the resulting QLD1516/2021 isolate sequence and remained stable in the passage 2 virus (Figure 3A). Whilst other recent SARS-CoV-2 sequences have reported a 6-nucleotide deletion in the same region, to our knowledge, this is the first 7-nucleotide indel reported resulting in the deletion of nucleotides 301–307 for the ORF7a gene. Putative translated protein sequences for ORF7a (Figure 3B) indicate that the 6-nucleotide indel results in the deletion of two amino acids (F and L) at residue positions 101 and 102, respectively, whereas the 7-nucleotide deletion in QLD1516/2021 confers an altered amino acid sequence (LLRQ) downstream of residue 100 followed by insertion of a premature stop codon. This strongly suggested that a truncated ORF7a protein product was being produced by QLD1516/2021 which may have defective functional characteristics. Also notable was the pairwise nucleotide sequence identity (99.0%) observed between the genomes of the recovered B.1.1.7 variants, QLD1516/2021 and QLD1517/2021. Indeed, the 1.0% difference in nucleotide identity was shown to be a result of the 7-nucleotide indel, and QLD1516/2021 shared 100% nucleotide identity with QLD1517/2021 across the remainder of the genome.

### 3.3. Kinetic Replication Curve Experiments

**Figure 4:**
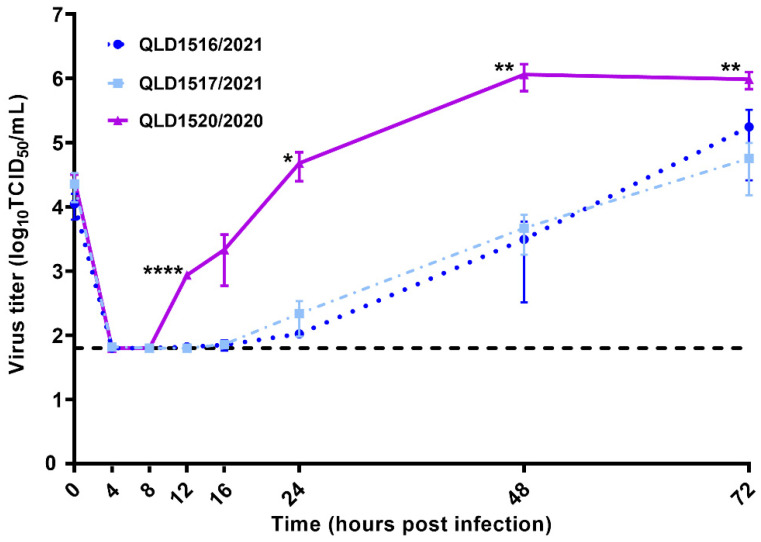
Kinetic replication curves (mean ± SD) of SARS-CoV-2 VOCs following infection of Vero E6 cells at a m.o.i. of 0.01. The black dotted horizontal line indicates the limit of detection (1.8 Log_10_ TCID_50_/mL) of the TCID_50_ assay. The virus titers at each time point were compared using one-way ANOVA and Tukey post-test. All comparisons between the two B.1.1.7 VOCs, QLD1517/2021 and QLD1516/2021, were not significant. Significant differences between QLD1520/2020 and the two B.1.1.7 VOCs are denoted as * *p* < 0.05, ** *p* < 0.01, and **** *p* < 0.0001.

The viral replication kinetics of the SARS-CoV-2 VOCs were investigated using Vero E6 cells, and the resulting replication curves of each of the viruses were distinctly different (Figure 4).

After an initial eclipse phase, QLD1520/2020 demonstrated a notably increased rate of replication between 8 and 24 h p.i., before peaking at 48 h p.i. Conversely, replication of QLD1517/2021 and QLD1516/2021 were delayed, and measurable titers in all three replicates of each of the viruses were not consistently obtained until 16 and 24 h p.i., respectively. At 12, 24, 48, and 72 h p.i., QLD1520/2020 produced significantly higher (*p* < 0.05) viral titers compared with either QLD1516/2021 or QLD1517/2021. Whilst replication of the QLD1516/2021 isolate was further delayed compared to QLD1517/2021, a more rapid rate of replication was observed between 24 and 72 h p.i. This resulted in QLD1516/2021 achieving similar titers at 48 h p.i. and slightly higher titers at 72 h p.i, compared to QLD1517/2021, although these differences were not significant (*p* > 0.05). Of note, NGS of viruses harvested at 72 h p.i. later confirmed retention of the previously identified 7-nucleotide deletion in the QLD1516/2021 ORF7a gene.

There was no detectable CPE in any of the VOC cultures by 24 h p.i. At 48 h p.i., low-level CPE was observed in both QLD1516/2021 and QLD1517/2021 cultures, being slightly more advanced for the latter isolate. By comparison, advanced CPE with major cell clearing was noted in the QLD1520/2020 culture at 48 h p.i., preceding a decline in its replication observed at 72 h p.i. (Figure 4). CPE continued to progress for both QLD1516/2021 and QLD1517/2021 at 72 h p.i., and although slightly more advanced for the QLD1517/2021 isolate, neither gross CPE (with extensive cell clearing) nor a decline in viral titers had yet commenced for either virus at this time point.

## 4. Discussion

Unlocking and systematically mapping the replication, evolutionary, and host–response dynamics of SARS-CoV-2 is fundamentally crucial for public health disease management and formulating improved vaccination, antiviral therapies, and epidemiological control strategies. The alarming emergence and number of several new SARS-CoV-2 VOCs with notably higher transmission rates, together with growing concern of their potential to evade some immune mechanisms or antigenically challenge vaccines, has renewed efforts to prioritize NGS and metagenomic surveillance of SARS-CoV-2 genomes worldwide. In January 2021, South Africa closed 20 land borders for over a month due to a surge in COVID-19 cases linked to the VOC identified as B.1.351 [20]. Since then, the B.1.351 variant has become dominant in South Africa’s Eastern and Western Cape provinces and has spread to at least 60 other countries, including the UK, Austria, Norway, and Japan [21,22]. Similarly, it has been reported that the B.1.1.7 lineage has, until recently, been the most dominant circulating lineage in both the UK and the USA, and has spread to more than 30 countries [23,24]. Among a rapidly growing list, other newly identified variants such as the B.1.617 lineage (reported from India), P.2 lineage (reported from Brazil), and the P.3 lineage identified in the Philippines are variants under investigation (VUI). These viruses are under assessment as to whether they are more transmissible, pathogenic, or lethal compared to other strains [25].

With regards to known VOCs, transmission rates based on increased incidence of virus detection or disease within populations have been widely reported. Despite this notoriety, there is still only limited information regarding other biological aspects, such as their individual replication characteristics and phenotypes. To empirically assess replication characteristics of the VOCs from the RSA and the UK, we investigated their respective replication kinetics in Vero E6 cells via measurement of live virus titers. We observed striking differences in the replication dynamics of QLD1520/2020 (B.1.351 VOC) compared to QLD1516/2021 and QLD1517/2021 (B.1.1.7 VOCs), with QLD1520/2020 demonstrating a shorter eclipse phase and more rapid rate of replication until 48 h p.i., at which viral titers peaked and were significantly higher than those produced by either QLD1516/2021 and QLD1517/2021. These findings based on three VOC isolates provide preliminary evidence that the replication dynamics of B.1.1.7 and B.1.351 VOCs may be different. This data will assist future assessment of their respective phenotypes, which may include broader studies incorporating more isolates and/or other experiments which more specifically investigate infection and replication parameters.

There are also international reports indicating that the B.1.351 VOC, which carries the receptor binding domain N501Y S protein mutation, may be approximately 50% more transmissible compared to earlier detected SARS-CoV-2 strains [22]. Early molecular in silico studies investigating the N501Y mutation also indicate it may enhance the binding affinity of the S protein to the human angiotensin converting enzyme 2 (hACE2) receptor and potentially facilitate more efficient entry and proliferation of the virus in host cells [26]. Whether the replication dynamics of individual VOCs correlate with different rates of transmissibility has yet to be investigated and will require more complex in vitro and in vivo studies.

Whilst the vast majority of people infected with the B.1.351 VOC have not experienced serious illness [21], there is mounting evidence that the B.1.1.7 VOC is not only capable of higher transmission rates, but in some instances, may also cause more severe disease compared to pre-existing SARS-CoV-2 variants [6]. Investigation of the B.1.1.7-variant-associated mechanisms responsible for illness or mortality is outside the scope of the current study, and although we demonstrated that the B.1.1.7 VOCs replicated less rapidly in Vero E6 cells compared to the B.1.351 VOC, close monitoring of further B.1.1.7 VOC mutations across the entire genome, besides those highly publicized for the S protein, remain warranted.

The recovery of QLD1516/2021, the second isolate belonging to the B.1.1.7 lineage, which was characterized by a 7-nucleotide mutation in the ORF7a gene, allowed us to assess potential phenotypic ramifications of defective ORF7a gene translation on SARS-CoV-2 replication kinetics. Global genomic surveillance has shown that mutations in the SARS-CoV-2 ORF7a gene are frequent and C-terminal end sequences are highly variable. Resembling QLD1516/2021, other SARS-CoV-2 sequences have been reported with premature stop codons in the ORF7a gene likely resulting in the production of a truncated, defective ORF7a protein derivative. In a previous study, a B.1 lineage SARS-CoV-2 mutant carrying an innate 115-nucleotide deletion in the ORF7a gene (ORF7a^Δ115^, genomic sequence still under review by GISAID as stated in Supplementary Materials Table S1 of the publication [27]) demonstrated notable replication impairment in Vero E6 cells [27]. Interestingly, it was postulated that the observed replication defect might be attributable to an elevated host cell interferon (IFN) response to SARS-CoV-2, in particular a type III IFN-dependent response, given that Vero E6 cells do not express type I IFN genes [27]. Whilst consistent replication (viral titers were measurable from all three replicates at a given time point) of the mutated QLD1516/2021 isolate in our study was the most delayed and was observed at least 8 h after consistent replication of the unmutated QLD1517/2021 isolate, virus production was not significantly impaired by 48 h p.i. Interestingly, higher QLD/1516/2021 viral titers were obtained compared to QLD1517/2021 by 72 h p.i. In contrast to previous replication experiments using ORF7a^Δ115^ [27], this suggests that suppression of host cell immune responses by the 7-nucleotide QLD1516/2021 isolate may have only been partially defective and enough key ORF7a sequence elements were retained to support viral egress mechanisms, although not necessarily equally. We demonstrated that after the initial replication impairment, viral titers produced by the QLD1516/2021 mutant were ultimately comparable with those produced by QLD1517/2021 by 48 h p.i. We also observed less CPE in the QLD1516/2021 cultured Vero E6 cells, which would provide favorable conditions for the continuation of replication. Future studies will be required to establish the time required to reach maximum titers of each of these B.1.1.7 VOCs, and more specifically investigate the mechanisms contributing to the observed phenotype associated with the 7-nucleotide ORF7a deletion.

In 18 months, the SARS-CoV-2 pandemic has re-defined human susceptibility to pathogenic viruses on a global scale. The emergence of new SARS-CoV-2 variants is inevitable whilst the pandemic remains unrestrained, and with that comes the heightened risk of the appearance of more transmissible or deadly strains. Thus, the collection of representative SARS-CoV-2 isolates and more rigorous phenotypic investigations to appraise the viral and host-interactive relevance of observed mutations is equally important to the existing genomic surveillance efforts and should be updated as new SARS-CoV-2 variants are discovered.

## Data Availability

Original patient sample and corresponding SARS-CoV-2 isolate sequences are available on the GISAID database at https://www.gisaid.org/. (accessed on 11 March 2021).
